# Pretreatment glasgow prognostic score predicts survival among patients administered first-line atezolizumab plus carboplatin and etoposide for small cell lung cancer

**DOI:** 10.3389/fonc.2022.1080729

**Published:** 2023-01-20

**Authors:** Satoshi Wasamoto, Hisao Imai, Takeshi Tsuda, Yoshiaki Nagai, Hiroyuki Minemura, Yutaka Yamada, Yukihiro Umeda, Takayuki Kishikawa, Ayako Shiono, Yuki Kozu, Jun Shiihara, Ou Yamaguchi, Atsuto Mouri, Kyoichi Kaira, Kenya Kanazawa, Hirokazu Taniguchi, Takayuki Kaburagi, Koichi Minato, Hiroshi Kagamu

**Affiliations:** ^1^ Division of Respiratory Medicine, Saku Central Hospital Advanced Care Center, Saku, Nagano, Japan; ^2^ Department of Respiratory Medicine, Comprehensive Cancer Center, International Medical Center, Saitama Medical University, Hidaka, Saitama, Japan; ^3^ Division of Respiratory Medicine, Gunma Prefectural Cancer Center, Ota, Gunma, Japan; ^4^ Division of Respiratory Medicine, Toyama Prefectural Central Hospital, Toyama, Toyama, Japan; ^5^ Department of Respiratory Medicine, Jichi Medical University, Saitama Medical Center, Saitama, Saitama, Japan; ^6^ Department of Pulmonary Medicine, Fukushima Medical University, Fukushima, Japan; ^7^ Division of Respiratory Medicine, Ibaraki Prefectural Central Hospital, Kasama, Ibaraki, Japan; ^8^ Third Department of Internal Medicine, Faculty of Medical Sciences, University of Fukui, Eiheiji, Fukui, Japan; ^9^ Division of Thoracic Oncology, Tochigi Cancer Center, Utsunomiya, Tochigi, Japan

**Keywords:** atezolizumab plus carboplatin and etoposide, body mass index, Glasgow prognostic score, immune checkpoint inhibitor, neutrophil-to-lymphocyte ratio, small cell lung cancer

## Abstract

**Background:**

There are no established predictive biomarkers for the effectiveness of first-line atezolizumab plus carboplatin and etoposide therapy in patients with small-cell lung cancer (SCLC). Therefore, the current study aimed to investigate whether the Glasgow prognostic score (GPS), neutrophil-to-lymphocyte ratio (NLR), and body mass index (BMI) can predict the effectiveness of first-line atezolizumab plus carboplatin and etoposide therapy in patients with extensive-disease SCLC.

**Methods:**

We reviewed data from 84 patients who received first-line atezolizumab plus carboplatin and etoposide therapy for SCLC at nine Japanese institutions between August 2019 and May 2021. Further, we evaluated the prognostic value of the GPS, NLR, and BMI. The Kaplan–Meier and Cox proportional hazard models were used to examine differences in progression-free survival (PFS) and overall survival (OS). Moreover, the GPS, NLR, and BMI consisted of C-reactive protein and albumin concentrations, neutrophil and lymphocyte counts, and body weight and height, respectively.

**Results:**

The response rate was 72.6% (95% confidence interval: 63.0–82.1%). The median PFS and OS from the initiation of treatment were 5.4 (95% CI: 4.9–5.9) months and 15.4 (95% CI: 11.4–16.8) months, respectively. The GPS independently predicted the effectiveness of first-line atezolizumab plus carboplatin and etoposide treatment, as a favorable GPS (GPS 0–1) was correlated with significantly better PFS and OS rates compared to a poor GPS (GPS 2) (PFS: 5.8 vs. 3.8 months, *p* = 0.0005; OS: 16.5 vs. 8.4 months, *p*<0.0001).

**Conclusions:**

This is the first analysis to evaluate the association between the GPS, NLR, and BMI and the treatment effectiveness of survival among patients receiving first-line atezolizumab plus carboplatin and etoposide therapy for SCLC. Among patients receiving this treatment for SCLC, GPS was significantly associated with the PFS and OS rates, suggesting that GPS might be useful for evaluating therapeutic outcomes in these patients.

## 1 Introduction

Small-cell lung cancer (SCLC) comprises approximately 15% of all lung cancer cases. SCLC is an aggressive tumor characterized by a rapid doubling time, high proliferation fraction, and early progression of widespread metastases (1, 2). Approximately 70% of patients with SCLC have reached the extensive disease (ED) stage at diagnosis; this is a stage correlated with a poor prognosis (3). Until recently, the standard first-line treatment for patients with ED-SCLC was platinum and etoposide combination chemotherapy. Despite a median overall survival (OS) period of approximately 10 months, there has been no significant development in OS for over two decades (4, 5). ED-SCLC is a poor prognostic disease with a median progression-free survival (PFS) period of 4.3–5.7 months, median OS period of 7.5–10.9 months, and a 5-year survival rate of 2.8% (5, 6). Recently, immune-checkpoint inhibitors (ICIs) have improved survival in patients with ED-SCLC (7–10). Atezolizumab is a humanized monoclonal anti-PD-L1 antibody that inhibits PD-L1 engagement with PD-1 and B7.1 (11). The randomized phase III trial (IMpower133) demonstrated significantly better survival outcomes with atezolizumab plus carboplatin and etoposide (AteCE) treatment than with carboplatin and etoposide combination chemotherapy (7, 8). Additionally, in the CASPIAN trial, durvalumab, another PD-L1 antibody, improved the median OS rates compared to the placebo despite the absence of significant benefit in the median PFS (9, 10).

Patients with lung cancer are often diagnosed at an advanced stage with distant metastases; many patients at advanced stages show a systemic inflammatory response (SIR) and weight loss, which affect cancer cachexia (12, 13). Therefore, the cancer-related prognosis is evaluated with a variety of SIR-based scoring systems, such as the Glasgow prognostic score (GPS) and neutrophil-to-lymphocyte ratio (NLR). The GPS is a scoring system that constitutes serum C-reactive protein (CRP) and albumin concentrations (12). SIR-based markers, such as GPS, can presage the efficacy of ICIs, with NLR predicting the efficacy of ICIs in malignant melanoma (14–16), renal cell carcinoma (17), and non-small cell lung cancer (NSCLC) (18–20). GPS has been reported in several studies to be an independent prognostic marker for ED-SCLC (21–24); however, no analyses have yet assessed the association between the GPS and ICI treatment effectiveness of first-line AteCE therapy for patients with SCLC. Moreover, a previous report indicated that the body mass index (BMI) is a prognostic index for various cancers, and sarcopenia was related to poor survival outcomes in patients with NSCLC treated with ICI (25). Furthermore, the BMI is related to ICI therapeutic effectiveness in solid malignancies, including malignant melanoma, renal cell cancer, and NSCLC (26). Recently, a report analyzed the relationship between the BMI and ICI outcomes in NSCLC cases (27). However, to the best of our knowledge, no studies have assessed the association between the BMI and ICI treatment efficacy in patients with SCLC. Therefore, the association between the BMI and the effectiveness of ICIs in SCLC cases remains unknown. Hence, data regarding the association between the GPS, NLR, and BMI and the efficacy of first-line AteCE treatment for patients with SCLC are limited. Therefore, this study aimed to evaluate whether the GPS, NLR, and BMI could predict treatment effectiveness for first-line AteCE treatment in patients with SCLC.

## 2 Patients and methods

### 2.1 Study patients

The current study is retrospective in design. This analysis evaluated the clinical effectiveness of first-line AteCE treatment in 84 patients with ED-SCLC at nine Japanese institutions between August 2019 and May 2021. Ninety-eight patients were administered AteCE-based combination chemotherapy. Among them, 14 patients received chemotherapy as second- or third-line treatment. The SCLC was histologically classified using the 2015 World Health Organization system and the disease stage was evaluated using the Tumor-node-metastasis (TNM) staging system (version 8). The inclusion criteria were as follows: (1) cytologically or histologically diagnosed SCLC, inoperable disease stage III/IV, or postoperative recurrence; and (2) first-line chemotherapeutic regimen with AteCE combination. The patients received first-line chemotherapy with AteCE, and each patient had a censored event or death confirmed. All patients were assessed *via* systematic evaluation and standardized staging procedures before receiving treatment. TNM clinical stage was assessed based on the results of physical examination, chest X-ray, thoracic and abdominal computed tomography (CT), brain magnetic resonance imaging or CT, and bone scintigraphy or ^18^F-fluorodeoxyglucose positron emission tomography. Furthermore, we assessed patients’ medical records to review data on baseline patient characteristics and tumor response to AteCE treatment. Data from patients who received AteCE treatment were collected as previously described (28).

The study design was approved by the Institutional Ethics Committee of International Medical Center, Saitama Medical University (approval number: 2021-113). All procedures complied with the ethical standards of the institutional and/or national research committee and with the 1964 Declaration of Helsinki and its later amendments or comparable ethical standards. This article does not contain any animal studies performed by any of the authors. Because of the retrospective nature of this study, the requirement for informed consent was waived.

### 2.2 Treatment

No patient had previously received ICIs, including AteCE chemotherapy. The basic treatment regimen comprised atezolizumab (fixed dose of 1,200 mg intravenously on day 1 of each cycle), carboplatin (area under the curve of 4–5 min mg/mL intravenously on day 1 of each cycle), and etoposide (body surface area of 80–100 mg/m^2^ intravenously on days 1–3 of each cycle) for four to six cycles. Next, this was followed by maintenance atezolizumab administration every 3 weeks. In some patients, atezolizumab was added to carboplatin and etoposide therapy during treatment based on the attending physician’s decision. Additionally, a granulocyte colony-stimulating factor was administered as neutropenia prophylaxis at the discretion of the treating physician. Moreover, treatment was discontinued on development of the progressive disease, observation of irreversible toxicity, or withdrawal of patient consent to treatment.

### 2.3 Treatment effectiveness assessment

Serum CRP and albumin concentrations were measured on the day or day preceding AteCE treatment administration. The GPS was categorized into three groups based on the combination of CRP value and albumin concentrations as follows: GPS of 0, 1, and 2 points included patients with a CRP level <1.0 mg/dL and albumin level ≥3.5 mg/dL, CRP increased or albumin decreased, and CRP level ≥1.0 mg/dL and albumin level <3.5 mg/dL, respectively. NLR was regarded as the ratio of absolute neutrophil and absolute lymphocyte counts. We set the cutoff value of the NLR at 5.0, considering a cutoff value of 4.91 (area under the curve: 0.593; sensitivity: 77.0%; specificity: 44.4%) based on the receiver operating characteristic curve analysis for OS (18, 29). Patients were classified into two groups based on their NLR levels: low (<5.0) and high (≥5.0). The BMI was calculated at treatment initiation as the weight (kg) divided by the height squared (m^2^). We analyzed the possible relationship between the BMI and AteCE efficacy using a BMI cutoff of 22.0 kg/m^2^, an ideal BMI in the Japanese population (30) (high BMI: ≥22.0 kg/m^2^; low BMI: <22.0 kg/m^2^).

Treatment response was assessed as the best overall response and maximum tumor shrinkage. Additionally, radiographic tumor responses were assessed according to the response evaluation criteria in solid tumors (RECIST), version 1.1 (31). PFS was calculated from day 1 of AteCE therapy until disease progression or death. OS was calculated from day 1 of AteCE therapy until death owing to any reason or censored on the last consultation date.

### 2.4 Statistical analyses

We adopted Fisher’s exact test and Welch’s t-test for categorical and continuous variables, respectively. We applied the Cox proportional hazards model with a stepwise regression procedure to identify factors that predicted PFS and OS. Hazard ratios (HR) and 95% confidence intervals (CI) were estimated. Univariate and multivariate logistic regression analyses were performed based on the different outcome variables. The Kaplan–Meier method was used to estimate survival as a function of time, and survival differences were analyzed using the log-rank test. Statistical significance was set at a two-tailed *p*-value ≤0.05. All statistical analyses were conducted using the JMP software for Windows, version 11.0 (SAS Institute, Cary, NC, USA).

## 3 Results

### 3.1 Patient characteristics and treatment efficacy

Overall, 84 patients were examined. **Table 1** presents the characteristics of 84 patients. We enrolled 70 male (83.3%) and 14 female (16.7%) individuals, with a median age of 71 (range, 43–89) years. The scores of the Eastern Cooperative Oncology Group (ECOG)-performance status (PS) were 0–1 and 2–3 points for 70 (83.3%) and 14 patients (16.7%), respectively. All patients had SCLC except for one patient with combined small cell carcinoma. There were 80 patients (95.2%) with stage III–IV SCLC. The median BMI was 22.0 (range, 14.1–32.8) kg/m^2^. Atezolizumab was added to carboplatin and etoposide in 16 patients. The median number of cycles of atezolizumab maintenance treatment was two (range, 0–24). **Table 2** shows the tumor response. Consequently, in the overall cohort, the overall response rate was 72.6% (95% CI: 63.0–82.1), and the disease control rate was 86.9% (95% CI: 79.6–94.1).

**Table 1:** Patient characteristics.

Characteristics	Total patient (n=84)
Sex	
Male/female	70/14
Median age at treatment (years) [range]	71 (43–89)
Performance Status (PS)	
0/1/2/3/4	19/51/8/6/0
Smoking history	
Yes/No	81/3
Histology	
Small cell carcinoma/combined small cell carcinoma	83/1
Clinical stage at diagnosis	
III/IV/postoperative recurrence	7/73/4
History of postoperative adjuvant chemotherapy	
Yes/no	1/83
Intracranial metastases at initial treatment	
Yes/No	25/59
Liver metastases at initial treatment	
Yes/No	20/64
Bone metastases at initial treatment	
Yes/No	30/54
BMI (kg/m^2^)	
Median [range]	22.0 (14.1–32.8)
Prior palliative radiotherapy	
Yes/No	7/77
Prior curative intent chemoradiotherapy	
Yes/No	4/80
Intermittent administration of atezolizumab with combined carboplatin and etoposide	
Yes/No	16/68
Number of cycles carboplatin+etoposide(+atezolizumab) administered	
Median	4
Range	1–6
Number of cycles of atezolizumab maintenance therapy administered	
Median	2
Range	0–24
Starting dose	
CBDCA (AUC 5)+etoposide (100 mg/m^2^)	60
CBDCA (AUC 5)+etoposide (80-99 mg/m^2^)	7
CBDCA (AUC 5)+etoposide (<80 mg/m^2^)	2
CBDCA (AUC 4)+etoposide (100 mg/m^2^)	1
CBDCA (AUC 4)+etoposide (80-99 mg/m^2^)	13
CBDCA (AUC 3.5)+etoposide (70 mg/m^2^)	1
With or without G-CSF prophylaxis	
Yes/No	50/34
Reason for discontinuation of carboplatin+etoposide+atezolizumab administration*	
Progressive disease	7
Adverse events	4
Others	7
Steroid treatment for adverse events**	
Yes/No	9/75
Laboratory data, Median [range]	
CRP (mg/dL)	0.56 (0.01–19.8)
Albumin (g/dL)	3.7 (2.0–4.7)
Neutrophil (cells/μL)	4,948.5 (2,279–26,094)
Lymphocyte (cells/μL)	1,385 (476–3,944)
Pro-GRP (pg/mL)	794 (24.4–129,200)
Continuing administration of atezolizumab at data cutoff	9/75

*excluding atezolizumab maintenance therapy.

**excluding topical agents.

PS, performance status; BMI, body mass index; AUC, area under the curve; G-CSF, Granulocyte colony stimulating factor; CRP, C-reactive protein; Pro-GRP, Pro-gastrin-releasing peptide.

**Table 2:** Treatment response.

	Total (n=84)
Response	
Complete response	5
Partial response	56
Stable disease	12
Progressive disease	9
Not evaluated	2
Response rate (%) (95% CI)	72.6 (63.0–82.1)
Disease control rate (%) (95% CI)	86.9 (79.6–94.1)

CI, confidence interval.

### 3.2 Comparison of the GPS, NLR, and BMI

**Table 3** shows patient characteristics based on the GPS, NLR, and BMI. Consequently, the GPS values at the beginning of AteCE treatment were 0–1 (63 patients) and 2 (21 patients) points. Liver metastases, administration cycles of atezolizumab maintenance therapy, CRP level, albumin level, neutrophil count, lymphocyte count, and response rate were significantly correlated (*p*<0.05) with GPS values. In turn, the NLR values at the beginning of AteCE treatment were low (60 patients) and high (24 patients). Prior radiotherapy, the CRP level, albumin level, neutrophil count, and lymphocyte count were significantly correlated (*p*<0.05) with NLR values. The BMI at the initiation of AteCE treatment was low in 44 patients and high in 40 patients. Sex, administration cycles of atezolizumab maintenance therapy, and lymphocyte counts were significantly correlated (*p*<0.05) with the BMI.

**Table 3:** Results of the patient’s characteristics according to the GPS, NLR, and BMI.

Variables	GPS			NLR			BMI		
	0–1	2	*p*-value	Low (<5)	High (≧5)	*p*-value	Low (<22.0)	High (≧22.0)	*p*-value
Patients (n)	63	21		60	24		44	40	
Characteristics									
Sex									
Male/female	51/12	19/2	0.50	47/13	23/1	0.05	33/11	37/3	**0.04**
Median age at treatment (years) [range]	71 (43–89)	71 (50–79)	0.41*	71 (43–89)	70 (50–78)	0.50*	71 (43–89)	70.5 (50–83)	0.47*
Performance Status (PS)									
0–1/≧2	55/8	15/6	0.1	50/10	20/4	>0.99	35/9	35/5	0.38
Smoking history									
Yes/No	60/3	21/0	0.56	57/3	24/0	0.55	42/2	39/1	>0.99
Intracranial metastases at initial treatment									
Yes/No	19/44	6/15	>0.99	16/44	9/15	0.42	15/29	10/30	0.47
Liver metastases at initial treatment									
Yes/No	11/52	9/12	**0.03**	13/47	7/17	0.57	10/34	10/30	>0.99
Bone metastases at initial treatment									
Yes/No	22/41	8/13	0.79	23/37	7/17	0.46	16/28	14/26	>0.99
BMI (kg/m^2^)									
Median [range]	22.0 (17.0–32.8)	20.5 (14.1–26.3)	0.05*	21.9 (14.1–32.8)	20.6 (17.0–28.2)	0.19*	20.0 (14.1–21.9)	23.4 (22.0–32.8)	<0.0001*
Prior radiotherapy**									
Yes/No	8/55	3/18	>0.99	4/56	7/17	**0.01**	7/37	4/36	0.52
Administration cycles of atezolizumab maintenance therapy									
Median (range)	3 (0–24)	1 (0–5)	0.034*	3 (0–18)	1 (0–24)	0.44*	2 (0–11)	3 (0–24)	0.0297*
Laboratory data									
CRP (mg/dL)	0.24	3.57	<0.0001*	0.53	1.98	<0.0001*	0.62	0.45	0.20*
Albumin (g/dL)	3.8	3	<0.0001*	3.8	3.3	0.004*	3.7	3.8	0.32*
Neutrophil (cells/μL)	4,619	6,846	0.0002*	4,511.5	6,851	<0.0001*	4,906.5	5,008	0.27*
Lymphocyte (cells/μL)	1,450	1,055	0.021*	1,510	900	<0.0001*	1,158	1,500	0.011*
Pro-GRP (pg/mL)	559	1,050	0.58*	593.8	923	0.96*	570.6	851.2	0.42*
Tumor response									
Complete response	5	0		5	0		2	3	
Partial response	46	10		41	15		28	28	
Stable disease	5	7		8	4		10	2	
Progressive disease	5	4		5	4		3	6	
Not evaluated	2	0		1	1		1	1	
Response rate (%) (95% CI)	80.9 (71.2–90.6)	47.6 (26.2–68.9)	**0.004**	54	62.5 (43.1–81.8)	0.27	68.1 (54.4–81.9)	77.5 (64.5–90.4)	0.46
Disease control rate (%) (95% CI)	88.8 (81.1–96.6)	80.9 (64.1–97.7)	0.45	90.0 (82.4–97.5)	79.1 (62.9–95.4)	0.28	90.9 (82.4–99.4)	82.5 (70.7–94.2)	0.33

Fisher’s exact test.

*Welch’s t-test.

**including palliative radiotherapy and curative intent chemoradiotherapy.

Bold font indicates a statistically significant difference.

GPS, Glasgow prognostic score; NLR, neutrophil-to-lymphocyte ratio; BMI, body mass index; PS, performance status; CI, confidence interval; CRP, C-reactive protein; Pro-GRP, Pro-gastrin-releasing peptide.

### 3.3 Survival analysis

**Figure 1:**
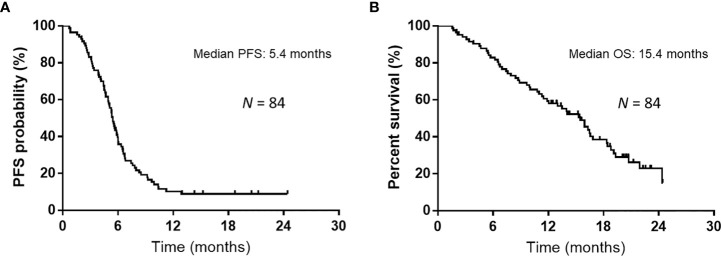
Kaplan–Meier curves for progression-free survival (PFS) and overall survival (OS). **(A)** The median PFS was 5.4 months among all 84 patients who received atezolizumab plus carboplatin and etoposide as first-line treatment. **(B)** The median OS was 15.4 months among all 84 patients who received atezolizumab plus carboplatin and etoposide as first-line treatment.

**Figure 2:**
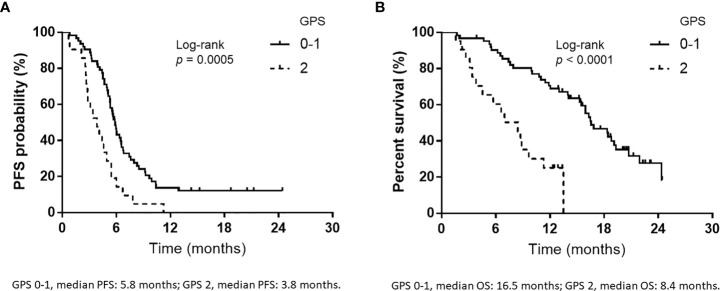
Kaplan–Meier curves for progression-free survival (PFS) and overall survival (OS) according to Glasgow prognostic score (GPS). **(A)** PFS according to GPS at the initiation of atezolizumab plus carboplatin and etoposide (GPS 0–1, median PFS: 5.8 months; GPS 2, median PFS: 3.8 months). **(B)** OS according to GPS at the start of atezolizumab plus carboplatin and etoposide (GPS 0–1, median OS: 16.5 months; GPS 2, median OS: 8.4 months).

Over a median follow-up duration of 12.9 (range, 1.5–24.4) months, the median PFS and OS intervals were 5.4 months (95% CI: 4.9–5.9 months) (**Figure 1A**) and 15.4 months (95% CI: 11.4–16.8 months) (**Figure 1B**), respectively. As of the data cutoff date of June 30, 2020, 54 of the 84 patients had died, and 30 were alive. **Table 4** presents the univariate and multivariate analyses of PFS and OS, respectively. The univariate analyses of PFS revealed significant correlations with liver metastases at initial treatment, prior radiotherapy, and the GPS. PFS was correlated with prior radiotherapy (HR: 0.31, *p* = 0.0029) and a GPS of 0–1 or 2 (HR: 0.42, *p* = 0.0036) in multivariate analyses. Furthermore, univariate analyses of OS showed significant correlations with liver metastases at initial treatment and GPS. Multivariate analyses showed that OS was correlated with a GPS of 0–1, or 2 (HR: 0.26, *p* = 0.0005). **Figure 2** depicts the survival curves (Kaplan–Meier analysis) for PFS and OS. A GPS of 0–1 was significantly associated with better PFS and OS than a GPS of 2 (*p*<0.05; **Figures 2A, B**). Patients with GPS 0–1 had a longer median PFS of 5.8 months than those with GPS 2, with a median PFS of 3.8 months (log-rank test, *p* = 0.0005; **Figure 2A**). Patients with GPS 0–1 had a longer OS of 16.5 months than those with GPS 2, with an OS of 8.4 months (log-rank test, *p<*0.0001; **Figure 2B**). **Supplementary Table 1** shows the number of treatment lines after AteCE therapy in the GPS 0–1 and 2 patient populations because GPS of 0–1 and 2 points were independent prognostic factors for PFS and OS, respectively.

**Table 4:** Univariate and multivariate analyses of PFS and OS.

Variables	Median PFS	Univariate analysis			Multivariate analysis			Median OS	Univariate analysis			Multivariate analysis		
	(months)	HR	95% CI	*p*-value	HR	95% CI	*p*-value	(months)	HR	95% CI	*p*-value	HR	95% CI	*p*-value
Sex														
Male/female	5.3/5.4	1.59	0.86–3.21	0.13				14.0/18.3	1.52	0.75–3.49	0.25			
Age														
<75/≧75 years	5.5/5.3	0.82	0.48–1.45	0.48				15.9/15.2	0.91	0.50–1.78	0.79			
Performance Status (PS)														
0–1/2–3	5.3/5.4	0.81	0.46–1.53	0.51	0.82	0.46–1.57	0.54	15.9/11.0	0.53	0.28–1.09	0.08	0.64	0.33–1.34	0.23
Smoking history														
Yes/No	5.4/4.6	1.02	0.37–4.20	0.96				15.4/6.5	0.90	0.27–5.55	0.89			
Intracranial metastases at initial treatment														
Yes/No	5.6/5.3	1.01	0.61–1.64	0.94				16.4/15.2	1.09	0.60–1.90	0.76			
Liver metastases at initial treatment														
Yes/No	4.9/5.5	1.86	1.08–3.08	**0.0257**	1.55	0.87-2.67	0.12	9.6/16.4	1.94	1.03–3.47	**0.04**	1.33	0.67–2.51	0.39
Bone metastases at initial treatment														
Yes/No	5.5/5.2	1.54	0.94–2.51	0.08				13.9/7.9	1.15	0.65–1.97	0.61			
Prior radiotherapy														
Yes/No	7.8/5.2	0.4	0.15–0.86	**0.0175**	0.31	0.11-0.69	**0.0029**	16.8/15.2	0.8	0.30–1.75	0.61			
GPS														
0, 1/2	5.8/3.8	0.41	0.24–0.70	**0.0017**	0.42	0.25-0.74	**0.0036**	16.5/8.4	0.23	0.11–0.46	**<0.0001**	0.26	0.13–0.55	**0.0005**
NLR														
Low (<5)/High (≧5)	5.5/4.4	0.87	0.52–1.50	0.61				15.9/12.0	0.72	0.40–1.38	0.31			
BMI (kg/m^2^)														
Low (<22.0)/High (≧22.0)	5.2/5.6	1.37	0.86–2.20	0.17				13.0/16.3	1.51	0.88–2.62	0.12			

Bold font indicates a statistically significant difference.

PFS, progression-free survival; OS, overall survival; HR, hazard ratio; CI, confidence interval; PS, performance status; GPS, Glasgow prognostic score; NLR, neutrophil-to-lymphocyte ratio; BMI, body mass index.

## 4 Discussion

This study examined the relationships of the GPS, NLR, and BMI with treatment effectiveness in patients treated with first-line AteCE therapy for SCLC. Multivariate analyses demonstrated that the GPS was independently correlated with PFS and OS, suggesting that the GPS may predict survival among patients treated with first-line AteCE therapy for SCLC. To the best of our knowledge, this is the first study to evaluate the relationship between the GPS, NLR, and BMI and survival among patients treated with first-line AteCE therapy for SCLC.

In this analysis, the group of patients with a GPS of 0–1 had a significantly higher response rate than those with a GPS of 2 points. Moreover, the GPS was significantly predictive of survival efficacy, such as PFS and OS. The GPS has been shown to have prognostic significance in SCLC independent of the disease stage and is conventionally adopted as a prognostic marker (21–24). Furthermore, the GPS has been associated with drug metabolism, adipokine levels, elevated cytokine levels, weight and muscle loss, and poor PS (13, 32–37). Furthermore, these factors are related to the host’s immune status and may influence the effectiveness of PD-1 blockade treatment. In this study, the associations between patient characteristics and the GPS were significantly correlated to liver metastases and administration cycles of atezolizumab maintenance therapy, indicating that the GPS is influenced by these clinical factors. Previous studies have investigated the GPS of first-line cytotoxic drug chemotherapy in patients with SCLC; however, no reports have investigated first-line ICI and cytotoxic drug chemotherapy in these patients.

Furthermore, the GPS is composed of the serum CRP value and albumin concentration levels, indicating that these laboratory data are frequently evaluated in daily clinical practice in most medical institutions. Additionally, multivariate analysis showed that the GPS, not the ECOG-PS, was independently associated with PFS and OS (**Table 3**). However, ECOG-PS has been used as a potent prognostic factor in clinical trials and clinical practice, and is a useful clinical indicator. Thus, evaluating the relationship between the GPS and ECOG-PS in large prospective and retrospective studies is necessary. The ECOG-PS is a subjective index-grading indicator that assesses the general condition of patients with malignant disease. In contrast, the GPS is an objective and has highly reproducible manner that classifies patients more precisely based on a three-index-scoring indicator. Therefore, the GPS may be more appropriate for pretreatment evaluations. In addition, GPS assessment is more objective than the usual prognostic factor of the ECOG-PS (38). A previous report demonstrated this from the era when ICI was not used; however, a previous report demonstrated that ECOG-PS did not show a significant difference in OS in patients with SCLC on multivariate analysis, in contrary to GPS (22). In reports on malignant lymphoma expected to respond to treatment, similar findings were reported. Especially, ECOG-PS showed no significant difference in PFS or/and OS in univariate and multivariate analysis, but GPS showed a significant difference (39–42). In summary, tumors that respond to treatment may have a poor PS at the beginning of treatment, but if the tumor responds to treatment, the PS at the beginning of treatment may have little effect on survival. In contrast, the treatment may be ineffective if the tumor has a GPS of 2 points, such as high CRP and low albumin levels at treatment initiation. Therefore, considering GPS in clinical practice for SCLC treatment may be reasonable.

Systematic reviews have reported the relationship between the NLR and clinical efficacy and outcome in patients with SCLC (43). A review article reported that the NLR is a prognostic factor; however, some reports have stated otherwise. The cutoff values have also been analyzed using various values. Several reports on the NLR for SCLC cases exist. For example, the NLR was identified as an independent negative prognostic factor for OS in patients with ED-SCLC at diagnosis (44). Pretreatment NLR may be useful in stratifying treatment approaches for patients with ED-SCLC (45). In this study, patient characteristics and the NLR were significantly correlated with prior radiotherapy, indicating that the NLR was influenced by clinical factors. Furthermore, it was not associated with either PFS or OS in patients with SCLC treated with first-line AteCE treatment. Although we analyzed a limited sample in our study, we could not find a significant relationship between the NLR and outcome after AteCE therapy for SCLC cases. However, we used the cutoff value that is frequently used in NLR studies and analyzed as previously described (46). In the future, we will investigate which cutoff value is optimal for NLR in a large population of patients with SCLC.

This analysis failed to detect BMI as a prognostic factor for SCLC. In previous reports, BMI evaluation and treatment efficacy for SCLC varied with BMI cutoff points, clinical stage, treatment, and analytical methods (43, 47–53). Most studies did not show a significant association between the BMI and SCLC survival prognosis (43, 47–49, 51, 53). Our result was consistent with those of previous analyses. However, a retrospective study including many patients suggested that a high BMI was correlated with prolonged PFS and OS following ICI therapy in patients with malignant melanoma (54). Other retrospective analyses on solid tumors, such as malignant melanoma, renal cell carcinoma, and NSCLC, demonstrated that the BMI is related to outcome of ICI therapy (26). Additionally, a correlation between the BMI in patients with NSCLC treated with ICI and survival outcomes has been observed (27). BMI is significantly correlated to the survival benefit of ICI treatment in patients with NSCLC treated with second-line or subsequent-line PD-1/PD-L1 blockade therapy. Moreover, patients with a high BMI have better outcomes. We previously reported that BMI independently predicted survival outcome, as patients with high BMI (BMI ≥21.4 kg/m^2^) demonstrated significantly better OS compared to those with low BMI (BMI <21.4 kg/m^2^) among patients with NSCLC expressing high PD-L1 who were administered first-line pembrolizumab monotherapy (46). Thus, there may be a difference in the relationship between the BMI and survival in NSCLC and SCLC cases. Additionally, in this study, the cutoff value for BMI was set at 22 kg/m^2^, but whether this is appropriate is a subject for future study, as it may be necessary to consider differences between different populations and ethnicities.

There are several limitations in this study. First, a retrospective study design, such as that in the current study, depends on subjective physician examinations, leading to variabilities in tumor response and PFS data. Second, the cutoff values for GPS, NLR, or BMI have not been established; there are various cutoff values indicated in previous reports, and we used previously reported cutoff values for the GPS, NLR, and BMI. Therefore, in the future, it is necessary to evaluate whether the results of this study are clinically reasonable in a larger patient cohort. Third, our sample size was limited, which may have affected our findings. However, this would be an inherent study limitation at most institutions, which do not have many patients with SCLC receiving first-line AteCE treatment. Thus, the potential significance of these sources of bias must be considered when interpreting our data. Fourth, the number of female patients in this study was small compared to the number of male patients, which may have affected the conclusion concerning sex difference. However, SCLC generally occurs more frequently in male, who are more likely to smoke, which inevitably results in a bias toward a smaller proportion of female.

In summary, GPS was identified as a significant predictor after ATeCE therapy for patients with SCLC. Further large-scale analyses are required to examine whether the results of our analysis can be generalized to other SCLC patient cohorts. Furthermore, whether the GPS can be considered a treatment effect modifier from our findings remains unclear. However, in the future, this clinical question could be addressed if larger studies on the effects of GPS subgroups in clinical trials of treatment including ICI are conducted.

## Data availability statement

The original contributions presented in the study are included in the article/**Supplementary Material**. Further inquiries can be directed to the corresponding author.

## Ethics statement

The study design was approved by the Institutional Ethics Committee of International Medical Center, Saitama Medical University. Written informed consent for participation was not required for this study in accordance with the national legislation and the institutional requirements.

## Author contributions

Conceptualization and methodology, SW and HI; formal analysis and data curation, HI and KyoK; project administration, visualization, and writing—original draft preparation, SW and HI; supervision, KyoK and HK; investigation and resources, TT, YN, HM, YY, YU, TKi, AS, YK, JS, OY, AM, KenK, HT, TKa, and KM. All authors contributed to the article and approved the submitted version.
