# Supplementary material for: Pretreatment glasgow prognostic score predicts survival among patients administered first-line atezolizumab plus carboplatin and etoposide for small cell lung cancer
Source: Front Oncol. 2023 Jan 20;12:1080729. doi: 10.3389/fonc.2022.1080729 (PMC9895374; doi:10.3389/fonc.2022.1080729)
Supplement: Supplementary file 1 [file Table_1.docx]

Supplementary Material

# Supplementary Tables

**Supplementary Table 1**. Status of treatment lines after progressive disease of the atezolizumab plus carboplatin and etoposide combination therapy for GPS 0–1/2 groups.

|  | Second-line | Third-line | Fourth-line | ≥Fifth-line |
| --- | --- | --- | --- | --- |
| GPS 0–1 (n=63) | 46 | 23 | 11 | 5 |
| GPS 2 (n=21) | 14 | 2 | 1 | 1 |

*Including continuing administration of atezolizumab at data cutoff
